# Nitrogen-Doped Porous Carbon from Biomass with Efficient Toluene Adsorption and Superior Catalytic Performance

**DOI:** 10.3390/ma15228115

**Published:** 2022-11-16

**Authors:** Jing Zhang, Jianwu Zou, Xiang Xu, Zhuang Li, Zheng Zeng, Liqing Li

**Affiliations:** 1 School of Energy Science and Engineering, Central South University, Changsha 410083, China; 2 Hunan Ecological and Environmental Affairs Center, Changsha 410014, China

**Keywords:** PtCo_3_ bimetal, carbon support, toluene oxidation, N-doped, toluene adsorption

## Abstract

The chemical composition and surface groups of the carbon support affect the adsorption capacity of toluene. To investigate the effect of catalyst substrate on the catalytic performance, two different plant biomasses, banana peel and sugarcane peel, were used as carbon precursors to prepare porous carbon catalyst supports (Cba, Csu, respectively) by a chemical activation method. After decorating PtCo_3_ nanoparticles onto both carbon supports (Cba, Csu), the PtCo_3_-su catalyst demonstrated better catalytic performance for toluene oxidation (T_100_ = 237 °C) at a high space velocity of 12,000 h^−1^. The Csu support possessed a stronger adsorption capacity of toluene (542 mg g^−1^), resulting from the synergistic effect of micropore volume and nitrogen-containing functional groups, which led to the PtCo_3_-su catalyst exhibiting a better catalytic performance. Moreover, the PtCo_3_-su catalyst also showed excellent stability, good water resistance properties, and high recyclability, which can be used as a promising candidate for practical toluene catalytic combustion.

## 1. Introduction

Growing volatile organic compounds (VOCs) from combustion and many different industry processes have aroused more concern for their damage to the atmospheric environment and human health [1,2]. As the most common volatile organic compound in the air, toluene is widely used as a solvent for paints, coatings, and adhesives and an important ingredient in various industrial and household products [3]. Long-term exposure to toluene may cause fatal harm to the human respiratory system, nervous system, and hematopoietic system. Therefore, there is an imminent need to develop technologies to dispel poisonous VOCs.

Many removal techniques are presented to improve air quality, including carbon adsorption [4], membrane separation [5], photocatalytic oxidation [6], non-thermal plasma oxidation [7], biological treatment [8], and catalytic oxidation [9,10,11]. Catalytic combustion is considered one of the most progressive and practical technologies for VOCs removal on account of its high efficiency, stability, and reliability. Supported noble metal catalysts (Pd and Pt) could achieve satisfactory catalytic activity under low temperatures. For example, Chen et al. synthesized a series of size-controllable Pt nanoparticles supported on ZSM-5. The Pt particles of 1.9 nm size could completely oxidize toluene at 155 °C [12]. The TiO_2_-supported 0.20 wt% Pt catalyst prepared by Wang et al. showed a high catalytic performance for toluene oxidation with T_50_ and T_90_ at 173 °C and 183 °C, respectively [13]. However, the high price of noble metal catalysts made them unable to be extensively used, and researchers were committed to finding alternatives with excellent performance. Fortunately, the addition of a second metal (Mn, Co, Fe, and Cu) to Pt made it reasonably priced, which may be a good alternative for developing oxidation catalysts [14,15].

Besides, the catalytic activity of supported catalysts was inextricably linked to the support. Generally speaking, catalyst supports could be divided into the following two categories [16,17]: one was active support, which had a positive catalytic effect on the oxidative combustion of VOCs, such as CeO_2_, Co_3_O_4_, MnO_x_, etc. [18,19,20]; the other was inert support, which on its own did not contribute to the catalytic removal of VOCs (such as C, Al_2_O_3_, molecular sieves, ceramics, etc.) [21,22,23]. However, the research on the role of supported catalysts and their substrates in catalytic oxidation remained elusive and needed to be further explored. Among the supports, biomass materials have become very promising support candidates due to their low cost, abundant pore structure, and large specific surface area and excellent adsorption capacity [24,25]. Zhao et al. prepared shaddock peel porous carbon with a specific surface area of 2398.74 m^2^ g^−1^ and the maximum adsorption capacity of the sample for methylene blue was 869.57 mg g^−1^ [26]. Li et al. prepared N-doped porous carbon from biomass lotus root as a precursor to becoming an efficient CO_2_ adsorbent [27].

In this work, two different biomass wastes, banana peel and sugarcane peel, were selected as carbon sources, zinc chloride and basic magnesium carbonate as double templates, and urea as a nitrogen source to prepare biomass porous carbon. Then PtCo_3_ nanoparticles were modified on these carbon supports, and the catalytic performance of the alloy was evaluated on the catalytic reaction testbed to explore the effects of carbon supports with different microstructures and chemical compositions on the performance of the catalyst. Furthermore, with the aid of multiple characterizations, the structure-property relationship and the potential mechanism were explored.

## 2. Experimental Methodology

### 2.1. Raw Materials

Biomass raw materials, banana peel and sugarcane peel, were gathered from Central South University in China. ZnCl_2_ (>98%), urea, toluene (95%), and hexane (>97%) were acquired from Sinopharm. Magnesium carbonate basic was purchased from Macklin. Hydrochloric acid (HCl, 36–38%) came from Xingkong, China. Chemicals used for the synthesis of catalysts such as cobalt acetylacetonate (Co(acac)_3_, 98%), platinum acetylacetonate (Pt(acac)_2_, 97%), oleic acid (AR), tetradecenediol (TDD, 90%), dichlorobenzene (>99.9%), benzyl ether (95%) and oleylamine (80–90%) were obtained from Aladdin. All the gases used in the experiments, such as nitrogen (99.99%), oxygen (99.99%), etc., were provided by High-Tech gas.

### 2.2. Materials Preparation

**Figure 1:**
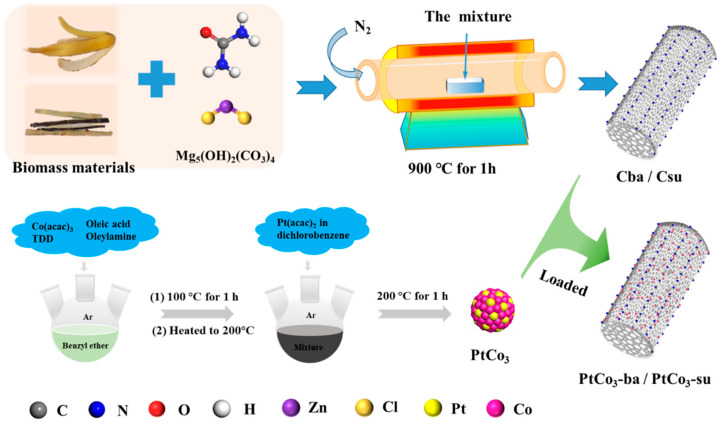
Diagram of PtCo_3_ catalyst synthesis method.

Both biomass raw materials were carefully washed with distilled water and then dried in a blast drying oven at 100 °C for 10 h. The dried biomass materials were crushed in a high-speed multifunctional crusher (800Y, Kemanshi, Jinhua, China) for 5 min. Each biomass, urea, basic magnesium carbonate, and zinc chloride were mixed at a mass ratio of 2:1:1:1 and ground in a mortar for 15 min. Then the mixture was heated to 900 °C in N_2_ atmosphere at a heating rate of 5 °C min^−1^ and carbonized at 900 °C for 1 h. The carbonized samples were washed in hydrochloric acid (80 mL, 2.0 M) for 12 h, after which they were cleaned with distilled water until the pH was neutral, and then dried at 60 °C for 12 h. The acquired samples were named Cba, Csu, respectively (Figure 1).

The preparation of PtCo_3_ catalysts supported on biomass carbon was prepared by organic phase reduction method. The mixture of 0.58 mmol Co(acac)_3_, 0.2 mL oleylamine, 0.2 mL oleic acid, 100 mg TDD and 15 mL benzyl ether in a three-necked flask was heated to 100 °C in Ar atmosphere and maintained for 1 h to dislodge moisture. When the temperature rose to 200 °C, 0.1 mmol Pt(acac)_2_ dissolved in 0.7 mL dichlorobenzene were added to reaction vessel and the reaction continued for 1 h. When the solution was cooled to room temperature, 25 mL of absolute ethanol was added to the solution and centrifuged at 10,000 rpm for 10 min to obtain PtCo_3_ nanoparticles (NPs). In order to decorate PtCo_3_ NPs on the carbon supports, 30 mL hexane and biomass carbon were mixed with PtCo_3_ NPs, then the compound was sonicated for 30 min. Then the samples were centrifuged (10,000 revolutions, 10 min). The PtCo_3_-ba and PtCo_3_-su samples were obtained after drying in a vacuum drying chamber at 60 °C for 18 h.

### 2.3. Materials Characterization

The morphologies of biomass carbons were visualized by scanning electron microscope (SEM, JSM-7900F, JEOL, Tokyo, Japan). The components and functional groups of the sample were identified by X-ray photoelectron spectroscopy (XPS, Thermo Scientific K-Alpha, Waltham, MA, USA). The crystal phase type of the samples was explored by X-ray diffraction (XRD, Rigaku SmartLab SE, Tokyo, Japan). Transmission electron microscope (TEM, Titan G260-300, FEI, Hillsboro, OR, USA) was used to characterize the surface morphology of catalysts. The surface area and pore structure of the samples were determined by using a JW-BK132Z static volumetric analyzer (Beijing JWGB Sci & Tech Co., Ltd., Beijing, China). The thermal stability of as-prepared samples was studied by thermal gravimetric analysis (TGA5500, TA, New Castle, DE, USA). The in-situ DRIFT test was carried out by FT-IR spectrometer (Thermo Scientific Nicolet iS50, Waltham, MA, USA). Additionally, the content of Pt and Co in the samples was quantified by inductively coupled plasma mass spectrometry (ICP-MS, Agilent 7700, Santa Clara, CA, USA).

### 2.4. Catalytic Tests

The activity and thermal stability of the catalyst were evaluated on the catalytic performance test platform. In total, 30 mg of catalyst and 1 g of quartz sand were mixed equably and placed into the quartz tube, which can be heated to a certain temperature by an external heating furnace, as shown in Figure S1. Moreover, all ventilation ducts were exteriorized with an insulation layer set to reduce heat transfer and eliminate environmental disturbances. The total reaction gas flow was 160 mL min^−1^, containing x ppm (x = 500, 1000, 1500) of toluene, 20% O_2_, and nitrogen, giving the gas hourly space velocity (GHSV) at 12,000 h^−1^. Toluene in different concentrations in a gas mixture was produced by using an N_2_ aerator through a bottle filled with pure toluene liquid, which was kept at 0 °C by a thermostatic water bath. The reactants and products were quantitatively analyzed by using an online gas chromatograph (GC-9860-5CNJ, Haoerpu, Nanjing, China) equipped with a flame ionization detector (FID) and TCD. Mass flowmeters and injection pumps were used to control gas concentrations when studying the effects of water vapor on the catalyst, respectively.

### 2.5. Calculations

Density functional theory (DFT) calculations were conducted via the Perdew–Burke–Ernzerh (PBE) of the generalized gradient approximation (GGA) method, and Materials Studio was the software used in calculations [28]. The convergence parameters set in the model optimization were as follows: gradient 2 × 10^−3^ Ha/Å, energy 1 × 10^−5^ Ha, and displacement 5 × 10^−3^ Å. The toluene adsorption energies onto the pure graphite and N-doped surface were derived in accordance with Equation (1) as follows:

*E_ads_ = E_surface+toluene_ − (E_surface_ + E_toluene_)* (1)

where *E_ads_*, *E_surface+toluene_*, *E_surface_*, *E_toluene_* represents the adsorption energy and total energy of adsorbent-toluene complex, adsorbent surface, and free toluene, respectively.

## 3. Results and Discussion

### 3.1. Catalytic Performance

**Figure 2:**
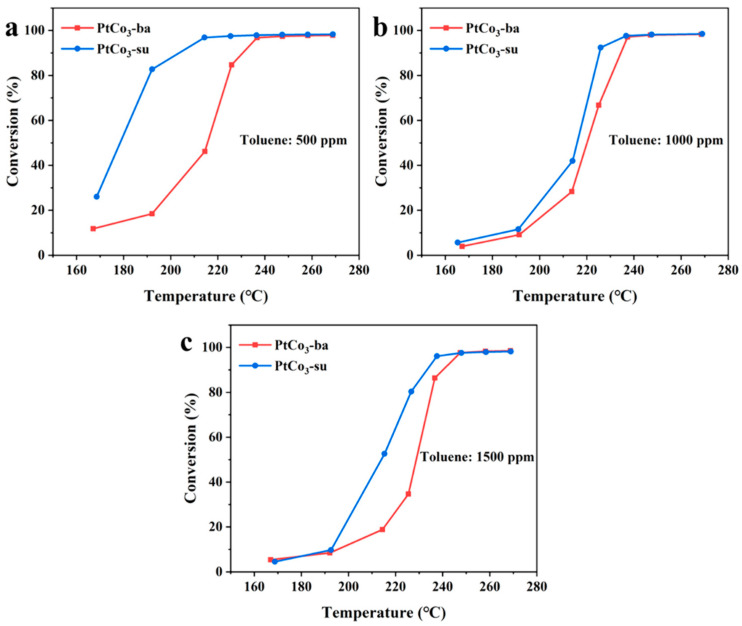
Conversion rates of as-synthesized samples at 500 ppm (**a**), 1000 ppm (**b**), 1500 ppm (**c**).

The catalytic performance of two PtCo_3_ catalysts for toluene combustion at 500, 1000, and 1500 ppm was assessed, and the results are shown in Figure 2. Two biomass carbon supports had no significant catalytic effect on the conversion of toluene (Figure S2) and possessed good thermal stability (Figure S3). When the toluene concentration was 500 ppm, toluene conversions of PtCo_3_-ba and PtCo_3_-su achieved 100% at 220 °C and 236 °C, respectively. The PtCo_3_-su and PtCo_3_-ba catalysts can completely remove toluene at 237 °C at a toluene concentration of 1000 ppm. Moreover, it was clear that the PtCo_3_-su sample can remove 91% of toluene at 226 °C, while that of the PtCo_3_-ba was 66%. When the concentration value of toluene was 1500 ppm, the PtCo_3_-su sample still showed a higher catalytic activity. The above results indicated that the PtCo_3_-su sample possessed an excellent catalytic performance for toluene at low reaction temperatures (Table S1).

**Figure 3:**
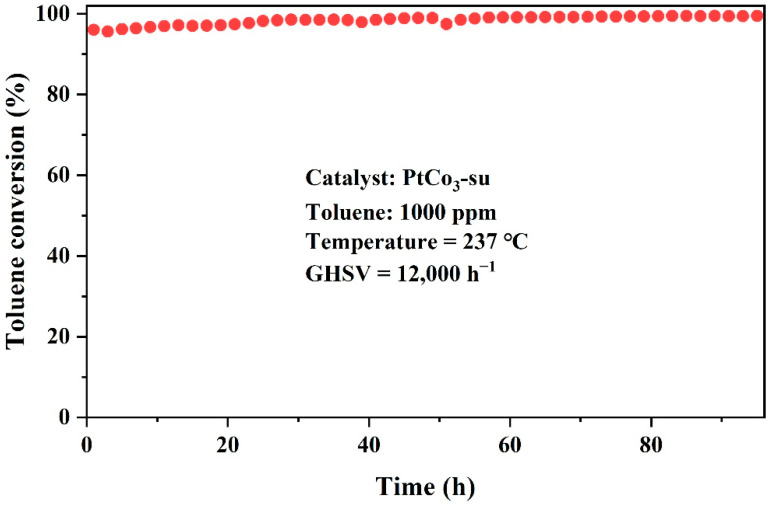
Reaction stability with time for toluene oxidation over PtCo_3_-su catalyst.

Catalytic durability was also a momentous issue in its holistic performance [29]. The stability of the PtCo_3_-su catalyst in consecutive reactions was examined at a reaction temperature of 237 °C and a GHSV of 12,000 h^−1^, and the results are shown in Figure 3. The catalyst maintained over 95% conversion to toluene, exhibiting an outstanding catalytic stability within a 96 h reaction time.

**Figure 4:**
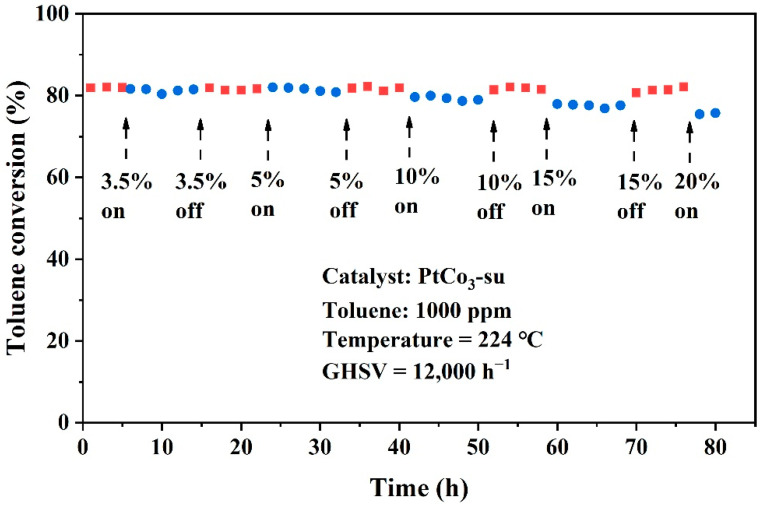
Reaction stability with time for toluene oxidation over PtCo_3_-su catalyst with 3.5 vol% to 20 vol% water vapor contents.

Numerous studies indicated that the existence of vapor had a negative impact on catalytic properties, leading to catalyst poisoning and deactivation. Inspired by Qi et al. [30], the effects of water on PtCo_3_-su catalyst performance were studied when the toluene conversion was 80% (Figure 4). The experiments were performed within 18 h as one cycle with an intermittent injection of water vapor. Obviously, as the water vapor content increased from 3.5% to 20%, the harmful effect of water vapor on the catalytic behavior became stronger. However, from the perspective of overall efficiency, it could be considered that there was no significant impact within 84 h. Meanwhile, the capability for toluene oxidation was quickly restored to a satisfactory value when the water vapor was cut off. The above-mentioned results make it clear that the PtCo_3_-su catalyst had excellent properties against water vapor.

### 3.2. Characterization of Biomass Carbon

**Figure 5:**
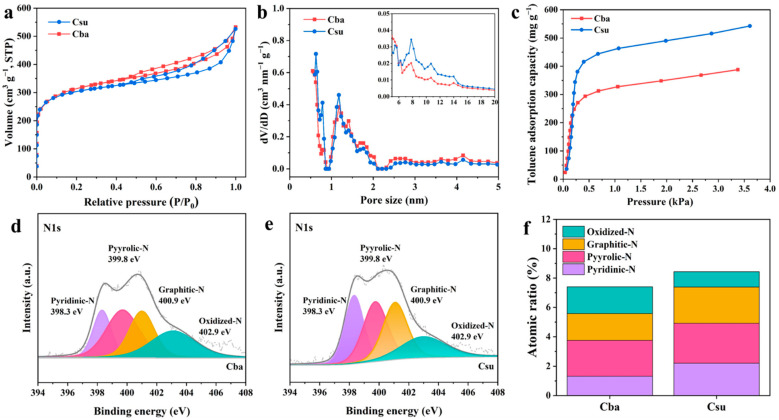
N_2_ adsorption and desorption (**a**), pore size distribution (**b**), toluene adsorption isotherms (**c**), N1s spectrum of the Cba, Csu (**d**,**e**), and atomic ratio of N species of Cba and Csu (**f**).

In terms of catalyst support, SEM was used to learn about the surface morphology of Cba and Csu. Both samples exhibited a rough surface and obvious pores (Figure S4). The N_2_ adsorption/desorption analysis was used to further explore the micropore structure of the prepared supports (Figure 5a).

The BET-specific surface area values and pore structure parameters of the supports were presented in Table 1. The two samples displayed a canonical type IV isotherm based on IUPAC classification [31,32,33], confirming the presence of mesopores. Additionally, the H3 hysteresis loop was observed in Cba and Csu. Figure 5b displays the pore size distribution (PSD) of two samples obtained from the non-local density functional theory (NLDFT) model [34,35]. The Csu sample had more micropores at 0.8–1.4 nm than the Cba sample. However, the Cba sample (1029 m^2^ g^−1^) possessed a higher BET-specific surface area than the Csu (960 m^2^ g^−1^).

Before loading alloy particles, the adsorption capacity of the support to toluene was explored, as shown in Figure 5c, which showed the order of PtCo_3_-su (542 mg g^−1^) > PtCo_3_-ba (387 mg g^−1^). Although the adsorption capacity of toluene was influenced by the BET-specific surface area and total pore volume, the micropore volume was the factor most closely related to it [36,37]. The micropore volume of the Csu was 0.377 cm^3^ g^−1^ while that of the Cba sample was 0.365 cm^3^ g^−1^. Besides, surface chemistry was also an important factor affecting the adsorption capacity [38]. As shown in Table 1, it was clear that the nitrogen content of Csu was higher than that of the Cba sample. Figure 5d,e shows the N1s spectrum and the four peaks obtained by its deconvolution. Four separated peaks were located at 398.3 eV, 399.8 eV, 400.9 eV, and 402.9 eV, respectively, referring to pyridinic-N, pyrrolic-N, graphitic-N, and oxidized-N [39]. Interestingly, the toluene adsorption capacity of Csu was approximately 1.4 times that of Cba, while the contents of pyridinic-N, pyrrolic-N, and graphitic-N showed a similar multiplier relationship. This could be interpreted in terms of the synergistic effect of micropore volume and nitrogen-containing functional groups. The microporous structure provided active sites for toluene adsorption, while the nitrogen functional group acted as the adsorption center and generated new active sites.

**Figure 6:**
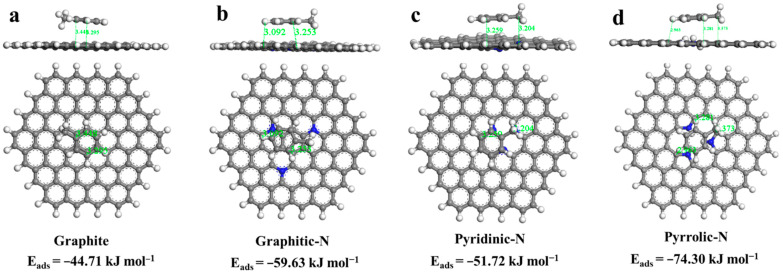
Optimized geometry and stable adsorption configurations of toluene on various nitrogen-containing functional groups, including pure graphite (**a**), graphitic-N (**b**), pyridinic-N (**c**), and pyrrolic-N (**d**).

To further investigate the intrinsic mechanism of the interaction between the N-containing functional group with toluene, N-containing functional groups were assessed by DFT calculations. Figure 6 shows the optimized geometry and the binding energies (BEs) of different nitrogen-containing functional groups in the carbon framework. It can be seen that the adsorption energies of all N-containing functional groups for toluene were higher than those on the pristine graphite model (−44.71 kJ mol^−1^). For three types of N-containing functional groups, the greatest BEs were obtained by pyrrolic-N (−74.30 kJ mol^−1^), followed by graphitic-N (−59.63 kJ mol^−1^) and pyridinic-N (−51.72 kJ mol^−1^). These results evidenced that the introduction of nitrogen-containing functional groups strengthened the electrostatic interaction, specifically referring to the π electrons formed on biochar that could form cation—π bonds via the electrostatic attraction on the benzene ring, thereby increasing the affinity of carbon materials with toluene molecules, in which the pyrrolic-N exhibited the strongest promoting effect [40,41]. This again confirmed that higher nitrogen content was one reason for the stronger adsorption capacity of Csu to toluene.

### 3.3. Characterization of Catalyst

**Figure 7:**
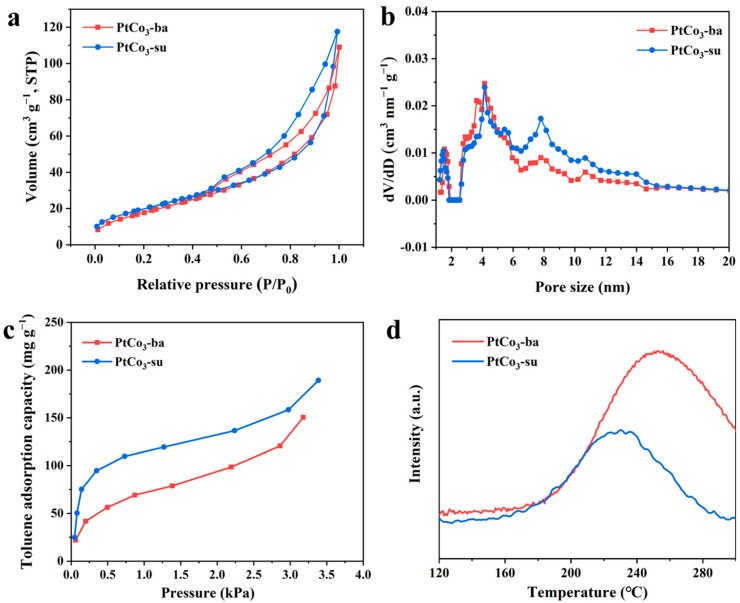
N_2_ adsorption and desorption (**a**), pore size distribution (**b**), toluene adsorption isotherms (**c**), and C_7_H_8_-TPD profiles (**d**) of catalysts.

As shown in Figure 7 and Table 2, both PtCo_3_-ba and PtCo_3_-su exhibited a similar specific surface area (68 m^2^ g^−1^ and 74 m^2^ g^−1^). The PtCo_3_-su obtained more mesopores of 6∼15 nm, presenting a larger total pore volume of 0.176 cm^3^ g^−1^, while the value for the PtCo_3_-ba was 0.147 cm^3^ g^−1^. The above results showed that PtCo_3_ loading caused a reduction in surface area, which mainly occupied pores with a diameter of 0.5–4 nm for two catalysts. The toluene adsorption and desorption properties of samples were determined by the static adsorption method and C_7_H_8_-TPD. Consistent with the previous adsorption on the supports, the PtCo_3_-su sample possessed a higher adsorption. Figure 7d illustrates that the peak center of PtCo_3_-su fell in a higher temperature area than that of PtCo_3_-ba. Such differences presented that PtCo_3_-su and toluene possessed a stronger interaction with each other than PtCo_3_-ba. This trend showed good consistency with the toluene conversion temperature of both catalysts, which suggested that the adsorption properties of toluene on the substrates had a significant impact on the catalytic activity.

**Figure 8:**
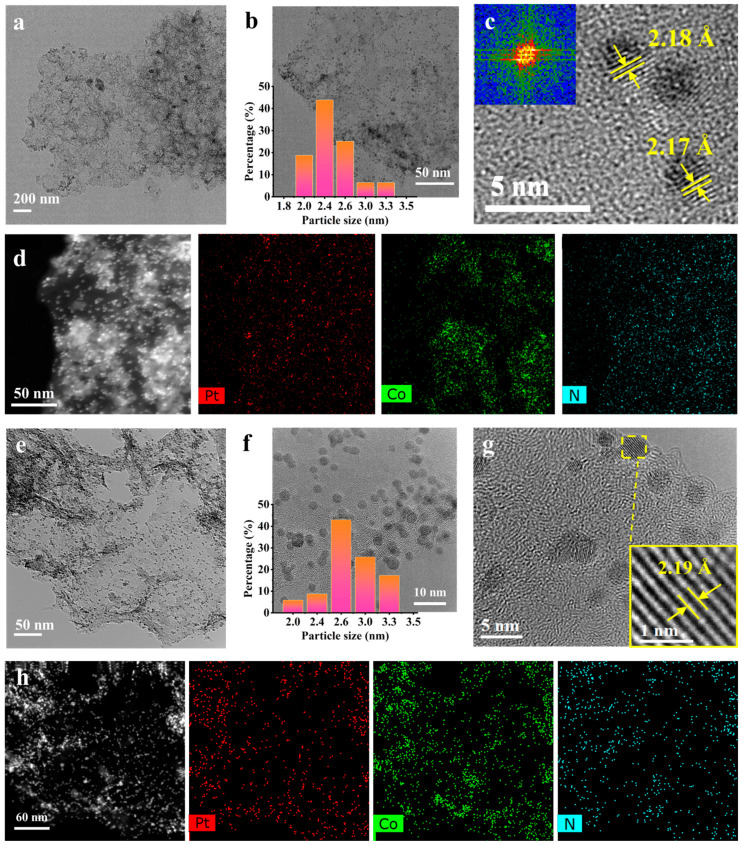
TEM images of PtCo_3_-ba (**a**,**b**), PtCo_3_-su (**e**,**f**). HRTEM of PtCo_3_-ba (**c**), PtCo_3_-su (**g**). Elemental mappings of PtCo_3_-ba (**d**), PtCo_3_-su (**h**).

The porous structure of the substrate, particle size, and structure of bimetallic PtCo_3_ nanoparticles were investigated intensively by TEM techniques. As shown in Figure 8a,e, the well-dispersed alloy nanoparticles could be observed from TEM images of PtCo_3_-ba and PtCo_3_-su, where the black dots represented the PtCo_3_ NPs and the gray transparent areas represented the support. Figure 8b,f showed the results of the size distribution analysis, which indicated that the PtCo_3_ were mostly in the form of tiny nanoparticles with a diameter of 2–3 nm. Figure 8c,g showed the images of Fourier fast transform (FFT) and inverse Fourier fast transform (IFFT) of HRTEM, indicating the presence of (111) crystal planes in the catalyst. The lattice distance of metal particles in the PtCo_3_-ba and PtCo_3_-su catalyst was 2.18 Å and 2.19 Å, which lay between the interplane distance of Pt (111) (2.27 Å) and Co (111) (1.98 Å), proving the alloy phase formation [40]. The alloy structural feature was further identified by elemental mapping (Figure 8d,h). The actual metal contents of PtCo_3_-ba and PtCo_3_-su were acquired via ICP-MS (Table 2). Additionally, the data from ICP revealed that the ratio of Pt to Co atoms in both PtCo_3_-ba and PtCo_3_-su was close to 37:84, implying PtCo_3_ was successfully synthesized.

**Figure 9:**
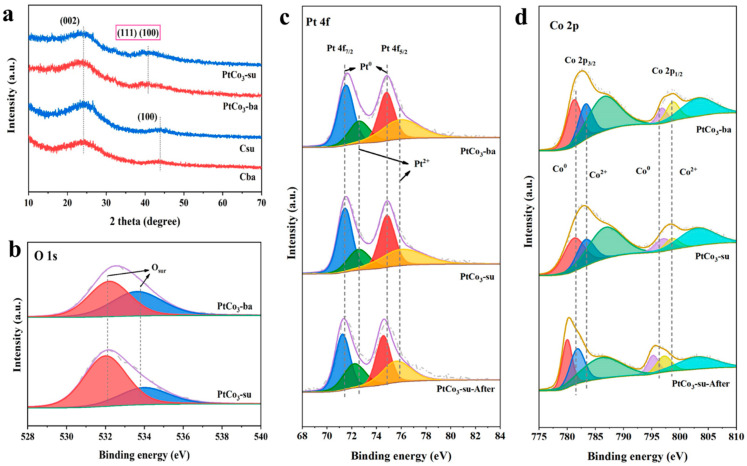
XRD patterns of samples (**a**), high-resolution XPS spectra of the O 1s (**b**), Pt 4f (**c**), and Co 2p (**d**) of as-prepared catalysts.

Figure 9a showed the XRD results of the support and two catalyst samples. For the former, the strong diffraction peak located approximately at 24.1° and the weak diffraction peak located at 43.8° corresponded to the (002) and (100) crystal facets of graphite crystals, respectively. After loading the metal, the characteristic peaks of Pt (111) (2θ of 39.5°) and Co (111) (2θ of 44.2°) were hardly observed in the two samples, and (002) crystal planes of graphite crystals were also hardly detected. The position of the peak changed to 42.1°, with a negative offset of graphite crystal and a positive shift of alloy, which may be the result of the combined action of (111) and (002) [30].

The XPS analysis was undertaken to find out the oxidation conditions of surface species, and the full XPS spectrum (Figure S5) of PtCo_3_-ba and PtCo_3_-su confirmed the presence of Pt, Co, C, N, and O. As indicated in Figure 9b, for the O 1s XPS spectra, two peaks at 532.2 and 533.8 eV corresponded to the surface oxygen species (O_ads_, O^2−^, $O_{2}^{2-}$, O^−^, or ${\mathrm{CO}}_{3}^{2-}$) with the absence of lattice oxygen [42,43]. Moreover, there was no large difference in the content of oxygen elements in the two catalysts. According to the results in Table 2, when the oxygen content of the PtCo_3_-ba sample was 4.562, the PtCo_3_-su was 4.314. The surface oxygen species would be involved in the later toluene oxidation process, which was an important element impacting the oxidation reaction.

Figure 9c,d showed the Pt 4f and Co 2p XPS spectra of the samples. For Pt 4f, the two peaks located at 71.62 and 74.68 eV corresponded to the Pt 4f_7/2_ and Pt 4f_5/2_ orbitals, respectively. The binding energy peaks of Pt 4f_7/2_ for Pt^0^ and Pt^2+^ on powdered metals were at roughly 74.9 and 75.9 eV, while Pt 4f_5/2_ were at 71.5 and 72.4 eV [44]. It was noticeable that the Pt species on two PtCo_3_ samples were mainly Pt^0^. As for Co 2p, the peaks of Co^0^ were located at 780.0 eV and 795.4 eV, and those of Co^2+^ are at 782.0 eV and 797.3 eV. Besides, the peaks located at 786.3 eV and 802.9 eV refer to the satellite peaks of Co 2p_1/2_ and Co 2p_3/2_ [45,46]. Moreover, the peak positions of Pt 4f and Co 2p of the PtCo_3_-su sample after the reaction were essentially the same as the fresh sample.

Besides, the PtCo_3_-su samples that had been tested for water resistance or thermal stability were also characterized by XPS (Figure S6). It turned out that among the tested samples, the XPS spectra of platinum and cobalt still displayed no apparent difference with fresh samples, further confirming their stable physicochemical properties.

### 3.4. Apparent Activation Energy

In order to deeply investigate the catalytic activity of two samples, a number of experiments were carried out to test the internal reaction speed. The amount of catalyst was reduced (15 mg) to reach a <20% removal rate of toluene with the goal of dislodging mass and thermal transfer restrictions [47]. The apparent activation energy (*Ea*) of PtCo_3_ samples for toluene oxidation was determined by the Arrhenius formula, as follows [48]:

*lnr* = −*Ea/R* + *lnA* (2)

where *r* is the reaction rate (mol s^−1^ g^−1^), *Ea* represents the apparent activation energy (kJ mol^−1^), *R* is a constant with the value 8.314 × 10^−3^, *T* refers to the absolute temperature in the reaction (K), while A is a pre-exponential factor. The reaction rate of toluene oxidation was obtained based on Equation (3) as follows [9]:

*r* = *(C_Toluene,In_* − *C_Toluene,Out_*)/*W* (3)

where *C_Toluene,In_* and *C_Toluene,Out_* represents the toluene concentration values at the inlet and outlet of the reaction device respectively (mol s^−1^), and *W* represents the weight of catalysts (g).

**Figure 10:**
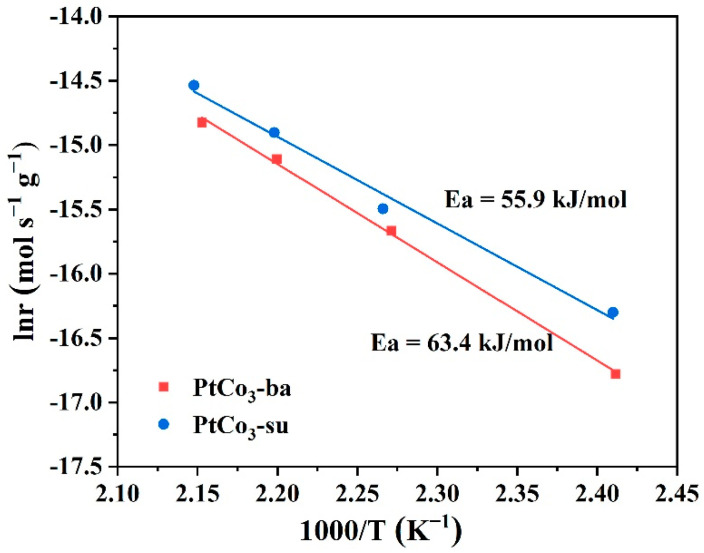
Arrhenius diagram of toluene oxidation on PtCo_3_-ba and PtCo_3_-su.

Figure 10 showed the Arrhenius diagram and *Ea* values of two samples. The *Ea* value (55.9 kJ mol^−1^) of the PtCo_3_-su catalyst was relatively lower than that of PtCo_3_-ba (63.4 kJ mol^−1^), thereby exhibiting a more superior catalytic activity, which was consistent with the results in Figure 2.

### 3.5. Reaction Mechanism

**Figure 11:**
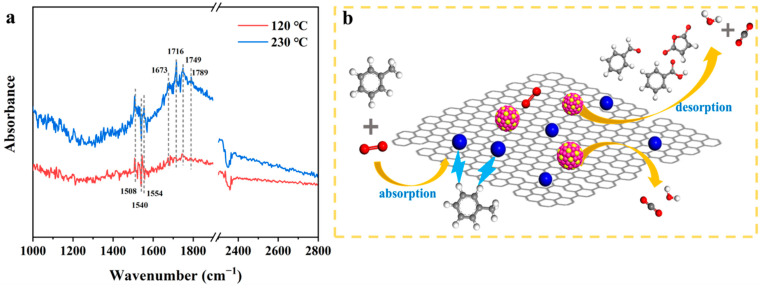
In situ DRIFTS spectrum of PtCo_3_-su (**a**) and probable toluene oxidation mechanism on PtCo_3_-su catalyst (**b**).

In situ DRIFTS were taken to elucidate the possible avenue of toluene oxidation, with the test consequences given in Figure 11a. The strong bands at around 1540 and 1508 cm^−1^ were caused by the skeletal vibration of aromatic rings [49]. The band at around 1749 cm^−1^ was relevant with C=O stretching vibration, which was a signal for the existence of species such as esters, aldehydes, or carboxylic acids [50]. Besides, the band at 1673 cm^−1^ can be assigned as the stretching vibration of the aldehydes, evidencing the production of benzaldehyde as the reaction proceeded [51]. The peak located at around 1789 cm^−1^ was assigned to COO^−^ stretching vibration, implying the presence of benzoate species. Along with the reaction, all intermediate components continued to be oxidized on the catalyst surface until they were finally converted into CO_2_ and H_2_O.

Figure 11b recapitulates the probable toluene oxidation mechanism on the PtCo_3_-su catalyst. Firstly, the involved toluene molecules were adsorbed on the surface of the PtCo_3_-su catalyst. With the increase in temperature, the surface-adsorbed oxygen species of bimetallic PtCo_3_ catalysts facilitated the disintegration of the C-H bond on methyl of toluene molecules. Then the products were continued to be oxidized to benzaldehyde and benzoic acid successively by surface active oxygen. Finally, the benzene ring was broken, and the molecules were quickly oxidized to H_2_O and CO_2_. Meanwhile, due to the consumption of surface-absorbed oxygen, the derived oxygen vacancies can be complemented by gas-phase O_2_, and thus active oxygen species will be formed [52]. The reaction proceeded until the toluene was completely removed from the gas. The excellent toluene adsorption capacity of the Csu sample enabled tight adsorption of toluene, benefiting the catalytic oxidation process of toluene. In a nutshell, the lower Ea and excellent adsorption property of the PtCo_3_-su sample were important reasons for the excellent catalytic performance.

## 4. Conclusions

In summary, two PtCo_3_ catalysts supported on two different kinds of carbon supports have been prepared by organic phase reduction and employed in toluene catalytic oxidative removal. Under identical external conditions, the PtCo_3_-su catalyst manifested better catalytic activity, which can accomplish the elimination of toluene at a temperature of 237 °C and achieve a conversion rate of 90% faster than PtCo_3_ NPs supported on Cba. Comparative characterizations revealed that higher N-containing levels of PtCo_3_-su were beneficial for the reactant adsorption, drastically boosting the catalytic activity. It was noticed that the PtCo_3_-su catalyst held remarkable stability, good water resistance properties as well as favorable recyclability. This work not merely provided an efficient catalyst for low-temperature toluene catalytic oxidation but also provided an effective strategy to improve the catalytic performance.

## Figures and Tables

**Table 1:** Structural properties and elemental compositions of biomass carbon support.

Samples	S_BET_ m^2^ g^−1^	V_total_ cm^3^ g^−1^	V_mic_ cm^3^ g^−1^	V_mes_ cm^3^ g^−1^	Micropore Percentage %	Average Pore Width nm	C at.%	O at.%	N at.%
Cba	1029	0.787	0.365	0.422	46.38	3.058	88.34	4.26	7.40
Csu	960	0.773	0.377	0.396	48.77	3.211	87.43	4.14	8.43

**Table 2:** Structural properties and elemental compositions of catalysts.

Samples	S_BET_ m^2^ g^−1^	V_total_ cm^3^ g^−1^	Pt	Co	O
PtCo_3_-ba	68	0.147	1.847	4.208	4.562
PtCo_3_-su	74	0.176	1.841	4.215	4.314

## Data Availability

Data are contained within the manuscript or Supplementary Material.

## Supplementary Materials

The following supporting information can be downloaded at: https://www.mdpi.com/article/10.3390/ma15228115/s1, Figure S1: Schematic diagram of catalyst performance test equipment; Table S1: Catalytic oxidation of toluene of bimetallic catalysts; Figure S2: Performance test results of two biomass carbon supports; Figure S3: TGA curve of two biomass carbon supports; Figure S4: SEM of Cba (a), Csu (b); Figure S5: XPS spectrum of PtCo_3_-ba and PtCo_3_-su; Figure S6: XPS spectrum of PtCo_3_-su after H_2_O and stabilization test: (a) Pt 4f and (b) Co 2p. References [53,54,55,56,57] are cited in the Supplementary Materials.
