# Supplementary material for: Nitrogen-Doped Porous Carbon from Biomass with Efficient Toluene Adsorption and Superior Catalytic Performance
Source: Materials (Basel). 2022 Nov 16;15(22):8115. doi: 10.3390/ma15228115 (PMC9698617; doi:10.3390/ma15228115)
Supplement: Supplementary file 1 [file materials-15-08115-s001.zip › materials-1987741-supplementary.pdf]

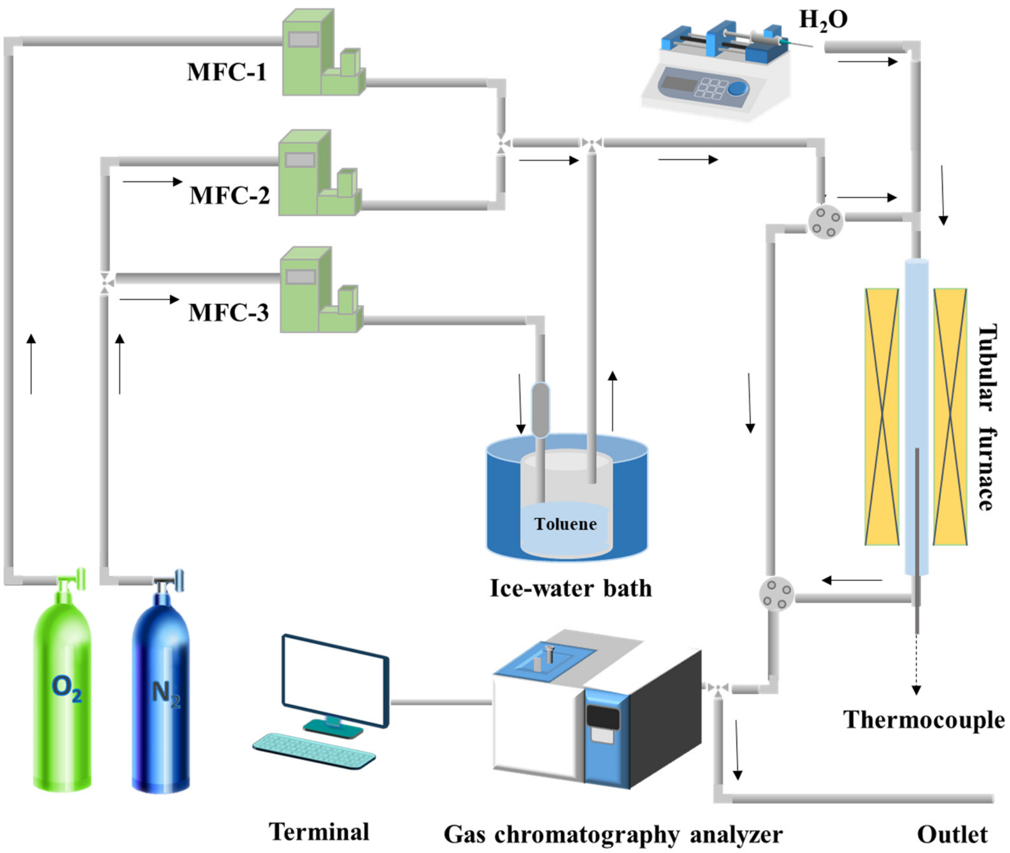

Figure S1. Schematic diagram of catalyst performance test equipment.

Table S1. Catalytic oxidation of toluene of bimetallic catalysts.

Samples	Temperature of complete oxidation of toluene (℃)	Ref.
$PtCo_3$-su	237	This work
10%Cr-Pt/ZSM-5	More than 255	[1]
Rh_1Cu_3/ZSM-5	More than 262	[2]
Ir/Ce(A)/Al_2O_3-H_2	310	[3]
Cu/Ag/ZSM-5	More than 300	[4]
$Cu_{0.2}Co$	257	[5]

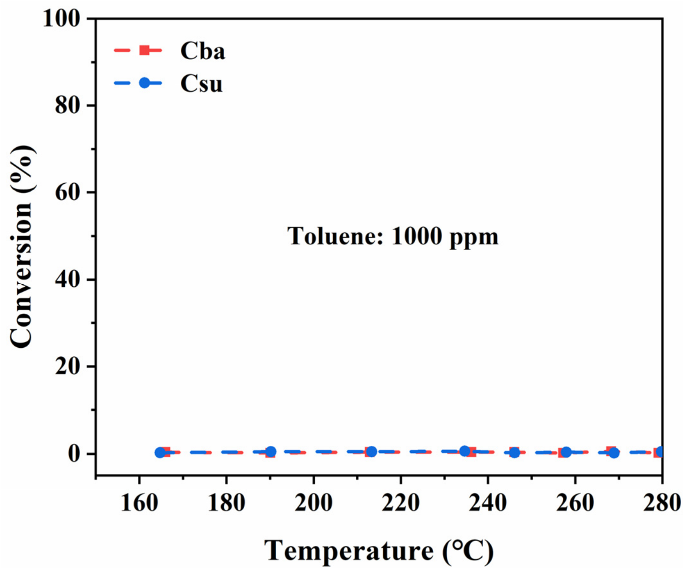

Figure S2. Performance test results of two biomass carbon supports.

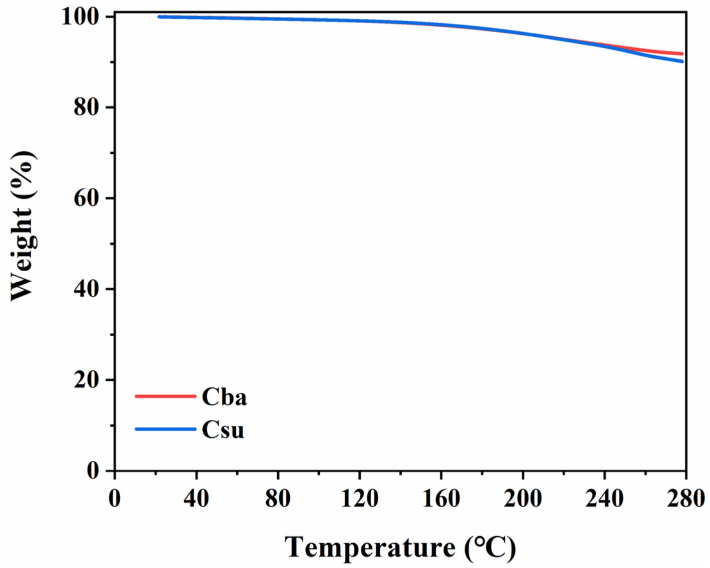

Figure S3. TGA curve of two biomass carbon supports.

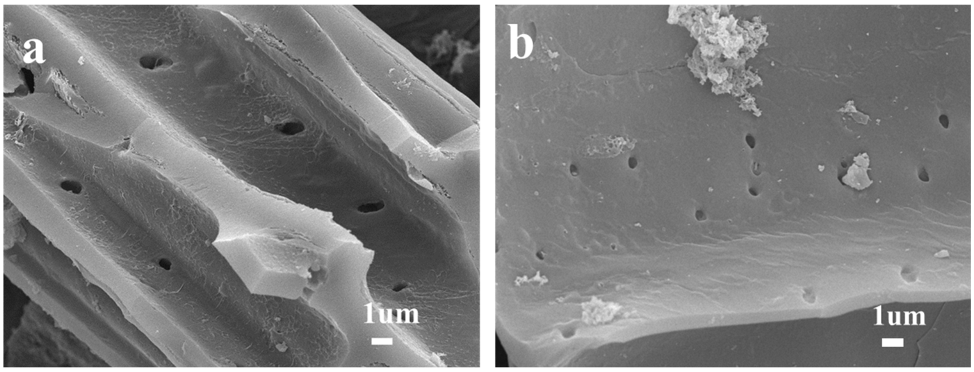

Figure S4. SEM of Cba (a), Csu (b).

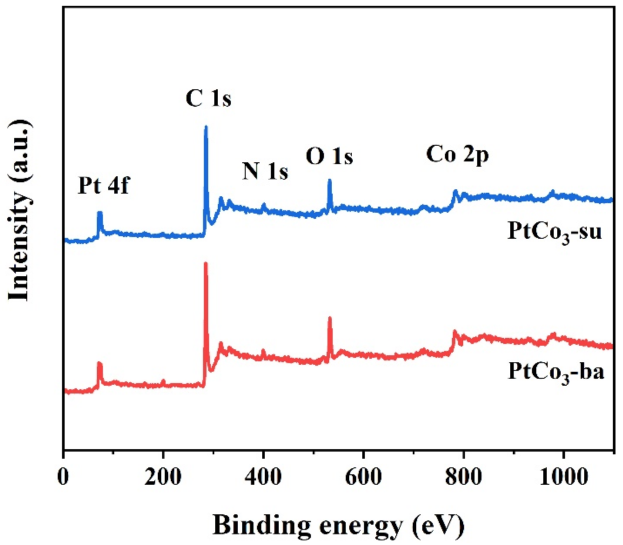

Figure S5. XPS spectrum of PtCo3-ba and PtCo3-su.

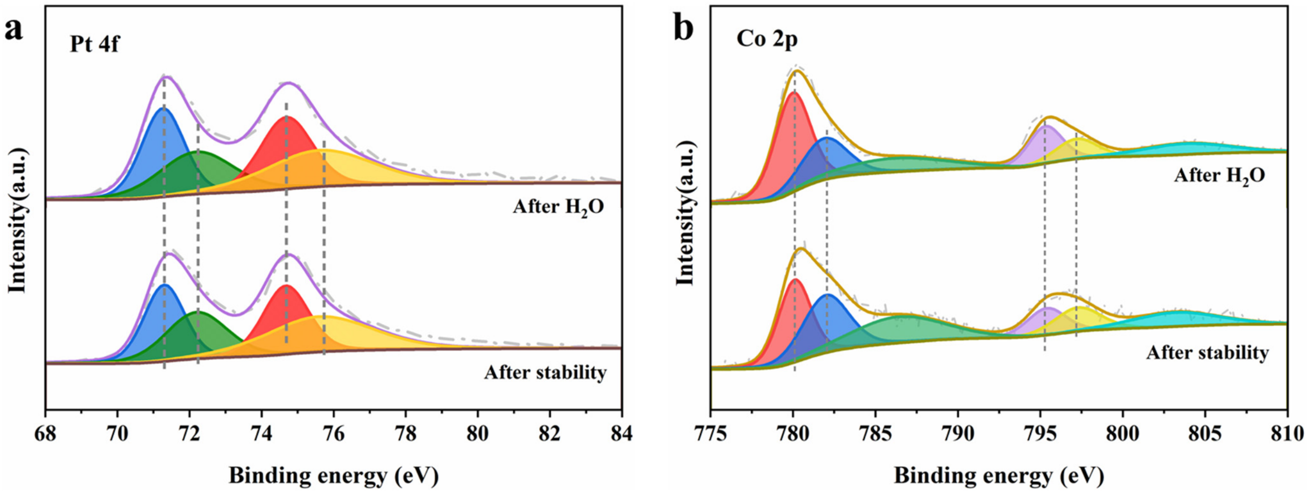

Figure S6. XPS spectrum of PtCo3-su after H_2O and stabilization test: (a) Pt 4f and (b) Co 2p.

References

[1] Shi Y, Wang J, Wang Y, Kong F, Zhou R. Synergistic effect in bimetal Cr-Pt/ZSM-5 catalysts with high activity for CVOCs low-temperature removal. J Environ Chem Eng 2022;10. https://doi.org/10.1016/j.jece.2022.107629.

[2] Yao S, Fang W, Wang B, Zeng Y, Chen L, Yan X, et al. Rh_1Cu_3/ZSM-5 as an Efficient Bifunctional Catalyst/Adsorbent for VOCs Abatement. Catal Lett 2021;152:771-80. https://doi.org/10.1007/s10562-021-03690-w.

[3] Wang Z, Zhang L, Ji J, Wu Y, Cai Y, Yao X, et al. Catalytic enhancement of small sizes of CeO_2 additives on Ir/Al_2O_3 for toluene oxidation. Appl Surf Sci 2022;571. https://doi.org/10.1016/j.apsusc.2021.151200.

[4] Niftaliyeva A, Karaduman A, Kalwar NH, Avci A, Pehlivan E. Synthesis of novel metal/bimetal nanoparticle-modified ZSM-5 zeolite nanocomposite catalysts and application on toluene methylation. Res Chem Intermed 2021;48:145-65. https://doi.org/10.1007/s11164-021-04597-2.

[5] Zhang W, Descorme C, Valverde JL, Giroir-Fendler A. Cu-Co mixed oxide catalysts for the total oxidation of toluene and propane. Catalysis Today 2022;384-386:238-45. https://doi.org/10.1016/j.cattod.2021.04.005.
